# Heated Air Delivery by Micro-Sauna: An Experimental Treatment Prototype Concept for Coronavirus Disease 2019

**DOI:** 10.7759/cureus.8162

**Published:** 2020-05-16

**Authors:** Ziyad O Knio, J. Alan Shelton, Tadhg O'Gara

**Affiliations:** 1 Department of Anesthesiology, University of Virginia, Charlottesville, USA; 2 Industrial Design, MIXXER Community Makerspace, Winston-Salem MIXXER Inc., Winston-Salem, USA; 3 Department of Orthopaedic Surgery, Wake Forest School of Medicine, Winston-Salem, USA

**Keywords:** covid-19, sars-cov-2, 2019-ncov, heated air, prototype

## Abstract

Coronavirus disease 2019 (COVID-19) has gained international attention as it poses a significant threat to global health. Currently, medical researchers are working to exhaust all strategies that may prove beneficial in combating this disease. Heat has been shown to destabilize other coronavirus strains in testing environments, and it has been hypothesized that heated air may destabilize viral pathogens in vivo as well. The present report describes the engineering of a micro-sauna prototype for the delivery of heated air. Concept formulation, process highlights, and the final prototype are all discussed. The prototype can deliver air heated to 80-90 degrees Celsius in a safe and tolerable manner. The goal of this technical report is to further encourage the study of heated air as a potential COVID-19 treatment.

## Introduction

Coronavirus disease 2019 (COVID-19), caused by severe acute respiratory syndrome coronavirus 2 (SARS-CoV-2), remains a global health emergency. As of April 17, 2020, the World Health Organization reports an aggregate of 2,074,529 total confirmed cases and 139,378 total deaths around the world [1]. The medical community is making considerable efforts to advance the understanding of this international emergency. Strategies for preventing COVID-19 transmission and infection have generally been classified as either pharmacologic initiatives or non-pharmacologic initiatives [2]. From a pharmacologic standpoint, antiviral agents, antimalarial agents such as chloroquine and hydroxychloroquine, and vaccination have received a great deal of attention [3-5]. Non-pharmacologic initiatives to date consist of health promotion strategies for disease mitigation and suppression, both within healthcare systems (transmission precautions, personal protective equipment), and for adoption by the general public (isolation and/or quarantine, social distancing, etc.) [6,7]. These strategies and this categorization, however, are neither exhaustive nor complete.

In accordance with the current mission to limit the spread and the impact of COVID-19, it is of paramount importance that medical researchers consider all available modalities that may prove useful in treating this disease. Heat has been shown to destabilize other coronavirus strains [8]. It has been hypothesized that heated air in vivo may destabilize viral pathogens as it does in vitro, though the evidence to support these claims is low quality [9]. Despite the lack of evidence, the negligible risk profile and the potential benefit warrant the investigation of heated air as therapy, whether for symptomatic benefit or, ideally, a reduction in viral shedding. The present report describes the engineering of a micro-sauna prototype for the delivery of heated air in a safe and tolerable manner. It is hypothesized that its administration may benefit subjects of varying degrees of illness severity, from those seeking primary prevention to patients who are critically ill. The goal of this technical report is to further encourage the study of heated air as a potential COVID-19 treatment.

## Technical report

Concept formulation

The prototype is intended to deliver air heated to 80-90 degrees Celsius (C). This temperature was selected as it is similar to that of dry saunas, and therefore should be well tolerated. Given that SARS coronavirus strain CoV-P9 has been shown to neutralize in a mimic-human environment after 90-, 60- and 30-minute exposure at 56, 67, and 75 degrees C, respectively, the selected 80-90 degrees C temperature range should theoretically have a great therapeutic window in vivo [8].

Process highlights

An initial attempt featured connecting a respirator mask to a bucket containing water heated to 80 degrees C. The most important take away from this experiment was that 80 degrees C water vapor delivered to the respiratory tract was not well tolerated in humans.

A subsequent experiment included porting a hairdryer into a 12-inch cube with a modified respirator mask connected. In this experiment, air temperatures dropped dramatically through the length of the hose. Moreover, the velocity with which air was delivered was not well tolerated.

It was concluded that an ideal prototype would feature the following: (a) heating elements larger than those in a hairdryer, (b) a concentrated area of heated air delivery to minimize the heat to the user’s face and body, (c) air delivery within a narrow temperature range, and (d) a short distance from the heat source to the respirator interface.

Final prototype

**Figure 1:**
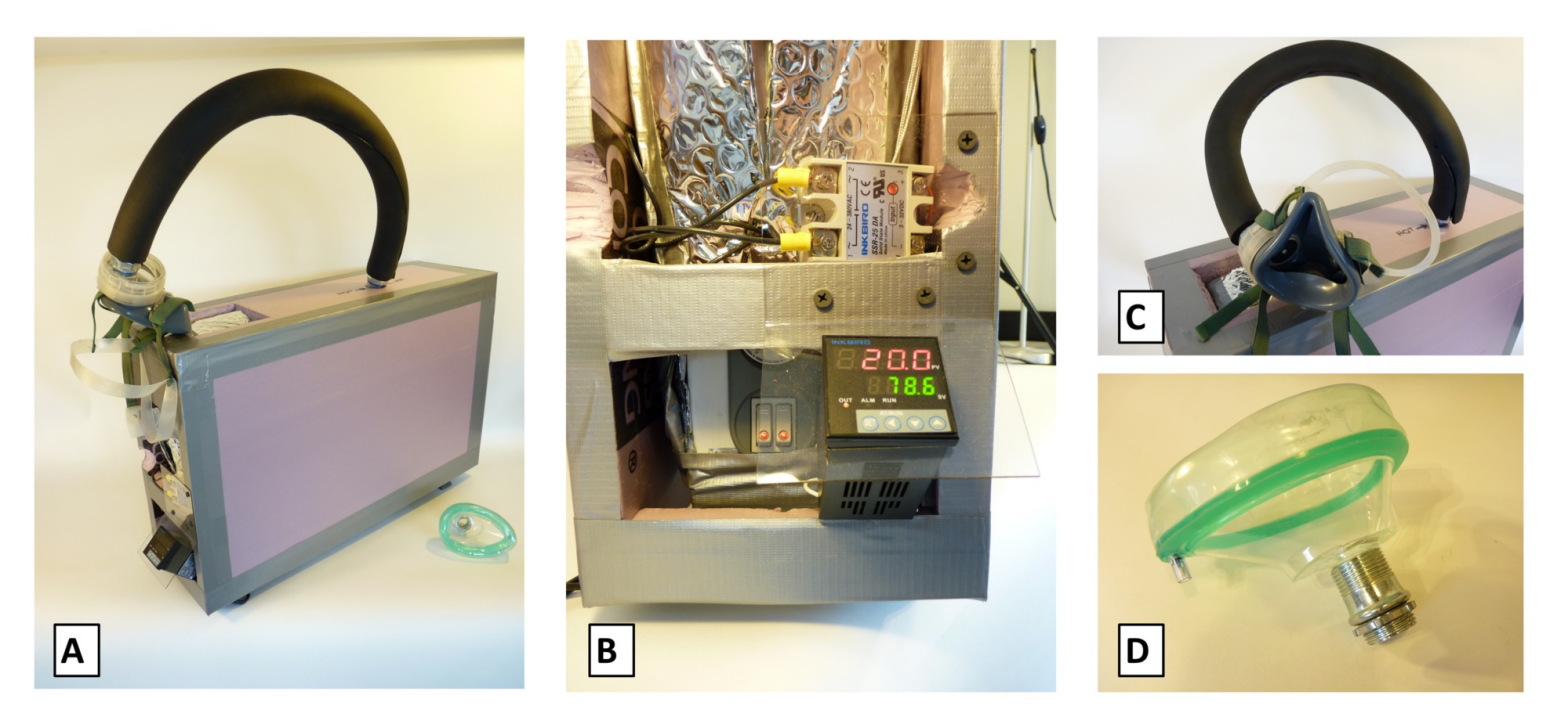
Micro-sauna final prototype (a) Prototype full assembly, and with component close-up of (b) heater and controller, (c) tubing and connectors, and (d) mask attachment.

The final prototype (Figure 1) features an electric heater, electric metallic tube (EMT) conduit connector, anesthesia mask (Medline Industries Inc., Northfield, IL, USA), and proportional-integral-derivative (PID) controller with thermocouple (Inkbird Tech. Co., Shenzhen, PRC).

An electric heater with resistive heat strips was selected over an infrared radiant heater because it did not require a fan to operate and it had a compact form factor. The internal circuitry was modified to bypass the tilt switch and the temperature sensor in order to use a microcontroller for more precise control of the heating elements. The vent holes on top of the heater were covered with a piece of sheet metal with a hole cut into it to allow connection of a fitting for a respirator interface.

The heater was wrapped in aluminum bubble wrap insulation, and then had a box built around it using one-inch Styrofoam insulation held together with nails and duct tape. A premium top-valve anesthesia mask was connected to a 3/4-inch EMT conduit connector that threaded into the sheet metal attachment site.

Finally, a PID controller with thermocouple was employed to manage the heating elements such that the temperature inside the micro-sauna could be maintained within a range of approximately 0.5 degrees C. Of note, the PID controller was successful, whereas an Arduino microcontroller with relays was not.

## Discussion

The present report demonstrates the feasibility of engineering a micro-sauna prototype for heated air delivery. The current prototype was designed for use as a home remedy, or for administration to hospitalized patients under investigation and non-critically ill COVID-19 patients. This strategy should be explored as a potential COVID-19 treatment, given that heat and ultraviolet irradiation have shown promise in eliminating SARS-CoV-P9 viral infectivity in near-human testing conditions, and may in fact reduce viral shedding in humans [8,9]. Even a reduction in symptom severity would be reason to administer heated air. There is a rapidly growing body of literature around COVID-19 prevention and/or treatment strategies, with several studies proposing rather unique approaches [10-12]. Despite the grand attention that COVID-19 has received, this is the first report to consider employing heated air as part of a COVID-19 treatment approach.

The motivation for considering heated air is based in an updated Cochrane Review by Singh et al. in 2017 [9]. Despite the review investigating heated, humidified air for the “common cold,” there is literature to support that this mechanism should translate to coronavirus strains [8]. Of the trials included in the Cochrane Review, only one defined viral titers as the primary outcome of interest [13]. During the five-day study period, there was no difference in mean rhinovirus titers, or proportion of subjects testing positive, between the cohort receiving 42-44 degrees C vapor (n=10) and the cohort receiving placebo (n=10). Such conclusions are limited by sample size. Further, 42-44 degrees C, while marginally greater than physiologic temperature, may have been a limiting factor. Duan et al. in 2003 observed SARS-CoV-P9 viral stability at 37 degrees C but non-infectivity at 56 degrees C and greater [8]. The moderate temperatures in the Cochrane Review may explain, in part, the inconclusive results in viral titer reduction and in the remaining studies examining symptom severity [9]. The micro-sauna prototype, with its delivery of air heated to 80-90 degrees C, is more likely to be successful.

The limitations of this report, as with any technical report, relate to its replicability and applicability. It is the authors’ hope that the detailed report on both the concept formulation and the micro-sauna’s construction will aid in replicability. The authors anticipate that the widespread use of micro-saunas may prove to be challenging, as there are many practical considerations around mass production of a medical device. For instance, the design for this prototype's subsequent model features a stainless steel exterior that may be sanitized and transported with inpatient beds such that the micro-sauna can be used in critical care settings. Additionally, it is unlikely that heated air can be tested as a stand-alone therapy for ethical reasons; its prescription as an adjunct to any indicated pharmacologic treatments may make interpretation of its independent effect challenging. Nevertheless, the successful development of this experimental treatment prototype may prove to be useful in future infectious disease research.

## Conclusions

Heated air delivery by a micro-sauna treatment prototype may prove beneficial in treating viral pathogens. Endpoints to consider include symptomatic benefit, viral shedding, length of hospital stay, and mortality rate. A micro-sauna delivering air heated to 80-90 degrees C can be feasibly engineered from an electric heater, EMT conduit connector, anesthesia mask, and PID controller with thermocouple. Following confirmation of its safety profile, this strategy should be explored as a potential COVID-19 adjunct treatment in varying patient care settings.
